# Propyl­ammonium 4,4,4-trifluoro-1-(naphthalen-2-yl)butane-1,3-dionate

**DOI:** 10.1107/S1600536811048318

**Published:** 2011-11-19

**Authors:** José A. Fernandes, Sofia M. Bruno, Isabel S. Gonçalves, Filipe A. Almeida Paz

**Affiliations:** a Department of Chemistry, University of Aveiro, CICECO, 3810-193 Aveiro, Portugal

## Abstract

The title salt, C_3_H_10_N^+^·C_14_H_8_F_3_O_2_^−^, constitutes the first organic crystal containing a residue of 4,4,4-trifluoro-1-(naphthalen-2-yl)butane-1,3-dione. The terminal –CF_3_ group is disordered over two locations [occupancy ratio = 0.830 (7):0.170 (7)]. Bond delocalization involving the two carbonyl groups and the α-carbon was observed. The crystal packing is mediated by several supra­molecular inter­actions, namely charged-assisted N—H⋯O hydrogen bonds, C—H⋯F and C—F⋯F short contacts and C—H⋯π inter­actions.

## Related literature

For general background to β-diketonates, see: Binnemans (2005 ▶); Bruno, Coelho *et al.* (2008 ▶); Bruno, Ferreira *et al.* (2008 ▶); Gago *et al.* (2005 ▶); Vigato *et al.* (2009 ▶). For coordination compounds having a naphtho­yltrifluoro­acetonate anion, see: Marandi *et al.* (2009 ▶); Ishida *et al.* (2007 ▶); Akhbari & Morsali (2007 ▶); Bruno *et al.* (2009 ▶); Fernandes *et al.* (2005 ▶, 2006 ▶); Lunstroot *et al.* (2009 ▶). For materials containing naphtho­yltrifluoro­acetonate, see: Bruno, Coelho *et al.* (2008 ▶); Bruno, Ferreira *et al.* (2008 ▶); Gago *et al.* (2005 ▶). For standard bond lengths determined by X-ray and neutron diffraction, see: Allen *et al.* (1987 ▶). For a description of the graph-set notation of hydrogen-bonded aggregates, see: Grell *et al.* (1999 ▶).
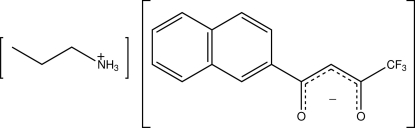


## Experimental

### 

#### Crystal data

C_3_H_10_N^+^·C_14_H_8_F_3_O_2_^−^
*M* *_r_* = 325.32
Monoclinic, 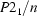

*a* = 16.1038 (11) Å
*b* = 5.7008 (4) Å
*c* = 17.2621 (11) Å
β = 91.497 (4)°
*V* = 1584.20 (19) Å^3^
*Z* = 4
Mo *K*α radiation
μ = 0.11 mm^−1^
*T* = 150 K
0.16 × 0.07 × 0.05 mm

#### Data collection

Bruker X8 KappaCCD APEXII diffractometer
Absorption correction: multi-scan (*SADABS*; Sheldrick, 1998 ▶) *T*_min_ = 0.982, *T*_max_ = 0.994
21307 measured reflections
2883 independent reflections
1958 reflections with *I* > 2σ(*I*)
*R*_int_ = 0.068

#### Refinement

*R*[*F* ^2^ > 2σ(*F* ^2^)] = 0.050
*wR*(*F* ^2^) = 0.117
*S* = 1.06
2883 reflections
246 parameters
42 restraints
H atoms treated by a mixture of independent and constrained refinement
Δρ_max_ = 0.20 e Å^−3^
Δρ_min_ = −0.21 e Å^−3^

### 

Data collection: *APEX2* (Bruker, 2006 ▶); cell refinement: *SAINT-Plus* (Bruker, 2005 ▶); data reduction: *SAINT-Plus*; program(s) used to solve structure: *SHELXTL* (Sheldrick, 2008 ▶); program(s) used to refine structure: *SHELXTL*; molecular graphics: *DIAMOND* (Brandenburg, 2009 ▶); software used to prepare material for publication: *SHELXTL*.

## Supplementary Material

Crystal structure: contains datablock(s) global, I. DOI: 10.1107/S1600536811048318/tk5018sup1.cif

Structure factors: contains datablock(s) I. DOI: 10.1107/S1600536811048318/tk5018Isup2.hkl

Additional supplementary materials:  crystallographic information; 3D view; checkCIF report

## Figures and Tables

**Table 1:** Selected bond lengths (Å)

O1—C2	1.268 (3)
O2—C4	1.256 (3)
C2—C3	1.377 (3)
C3—C4	1.417 (3)

**Table 2:** Selected short distance supra­molecular inter­actions (Å, °) *Cg* is the centroid of the C8–C14 ring.

*A*—*B*⋯*C*	*A*—*B*	*B*⋯*C*	*A*⋯*C*	*A*—*B*⋯*C*
**Strong hydrogen bonds**				
N1—H1*X*⋯O1^i^	0.95 (1)	1.98 (1)	2.871 (3)	155 (2)
N1—H1*Y*⋯O2^ii^	0.96 (1)	1.88 (1)	2.798 (3)	159 (2)
N1—H1*Z*⋯O1^iii^	0.95 (1)	1.98 (1)	2.835 (2)	148 (2)
N1—H1*Z*⋯O2^iii^	0.95 (1)	2.34 (2)	2.985 (3)	124 (1)
**C—H⋯F contacts**				
C14—H14⋯F1^iv^	0.95	2.66	3.305 (4)	126
C13—H13⋯F1^iv^	0.95	2.69	3.320 (4)	124
C13—H13⋯F3^iii^	0.95	2.64	3.337 (4)	130
**C—H⋯π contacts**				
C11—H11⋯*Cg*^v^	0.95	2.93	3.677 (3)	136
C17—H17*C*⋯*Cg*^vi^	0.98	2.87	3.786 (3)	157
**F⋯F contact**				
C1—F3⋯F3^vii^	1.30 (1)	2.78 (1)	3.409 (4)	108 (1)

Symmetry codes: (i) 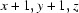; (ii) 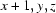; (iii) 
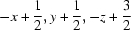
; (iv) 
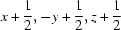
; (v) 
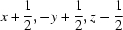
; (vi) 
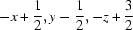
; (vii) 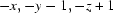.

**Fig. 1.**
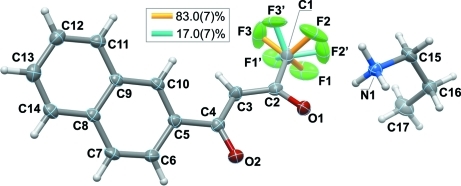
Molecular units (anion plus cation) composing the asymmetric unit of the title compound. Displacement ellipsoids are drawn at the 50% probability level and the atomic labeling is provided for all non-hydrogen atoms. Hydrogen atoms are represented as small spheres with arbitrary radius.

**Fig. 2.**
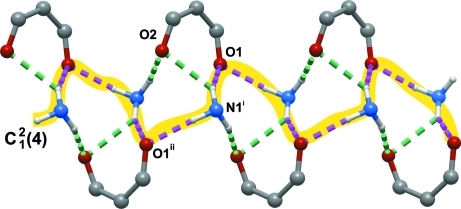
Hydrogen bonded supramolecular chains running parallel to the [010] direction of the unit cell. The N+—H···O- interactions composing the main spiral chain are represented by dashed pink lines and highlighted in yellow in the background. The N+—H···O- interactions located solely on the secondary spiral are represented as dashed green lines. For geometric details of the represented interactions see Table 2. Symmetry operations used to generate equivalent atoms: (i) 1/2 - x, -1/2 + y, 3/2 - z; (ii) -1/2 - x, 1/2 + y, 3/2 - z.

**Fig. 3.**
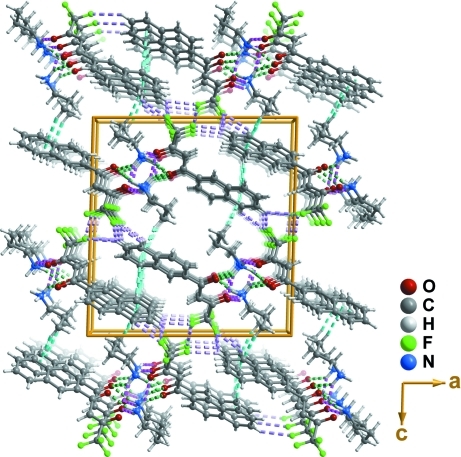
Crystal packing of the title compound viewed along the [010] direction. Only the F atoms corresponding to the most representative part are represented (F1, F2 and F3). Strong hydrogen bonds are represented following the colour scheme of Figure 2. Short distance interactions of the type C–H···F and F···F are represented as dashed violet lines. C—H···π interactions are depicted as dashed blue lines. For geometric details of the represented interactions see Table 2.

## supplementary crystallographic information

### Comment

β-Diketones are composed of two carbonyl groups separated by one carbon atom, the α-carbon. The conjugation of the two double bonds stabilizes the β-diketonate anion, which results from the loss of a proton of the neutral β-diketone. β-Diketonates are good chelating ligands, and under appropriate conditions might form mono- and polynuclear complexes, with *d*- and *f*-block elements (Binnemans, 2005). These types of complexes may find several direct applications, mainly in the fields of photoluminescence, NMR spectroscopy and biomedicine, due to their intrinsic optical and magnetic properties (Bruno, Coelho *et al.*, 2008; Bruno, Ferreira *et al.*, 2008; Gago *et al.*, 2005; Vigato *et al.*, 2009). 1-(2-Naphthoyl)-3,3,3-trifluoroacetone (HNTA) (systematic name: 1-(2-naphthyl)-4,4,4-trifluorobutane-1,3-dione) is a β-diketone which has been used in the preparation of a handful of coordination compounds with lead (Marandi *et al.*, 2009), transition metals (Ishida *et al.*, 2007; Akhbari & Morsali, 2007) and lanthanides (Bruno *et al.*, 2009; Fernandes *et al.*, 2005, 2006; Lunstroot *et al.*, 2009). Our group has been interested in the photoluminescence behaviour of the latter family of compounds. In this context we have prepared a handful of photoluminescent materials comprising HNTA residues and lanthanides (Bruno, Coelho *et al.*, 2008; Bruno, Ferreira *et al.*, 2008; Gago *et al.*, 2005). As part of our on-going research we have isolated the title compound, which represents to the best of our knowledge the first organic crystal containing HNTA residues.

The asymmetric unit of the title salt comprises one propylammonium cation (PrNH_3_^+^) and one naphthoyltrifluoroacetonate anion (NTA^-^) (Fig. 1). The +synclinal torsion angle N1—C15—C16—C17 of PrNH_3_^+^ has a value of 65.1 (3)°. The —CF_3_ group in NTA^-^ exhibits rotational disorder, which was modelled by the superposition of two different parts whose rate of occupancy was refined and converged to 0.830 (7) and 0.170 (7), respectively. Most atoms of this anion are placed in two planes: the naphthyl carbon atoms (C5 to C14) and C4 define plane A [largest deviation of 0.042 (2) Å for C4]; C1 to C4, O1, F2' and F3 define plane B [largest deviation of 0.049 (3) Å for C1]. The angle subtended by planes A and B is 39.0 (2)°, while the bond C4—O2 subtends an angle of 16.2 (3)° with plane B (*i.e.*, the bond is raised from the plane). Atoms O1, C2 to C4 and O2 are engaged in a system of delocalized bonds due to the proton transfer to propylamine. This feature is evident in the C—C and C—O distances which are intermediate between the expected values for single and double bonds (see Table 1 for geometric details; Allen *et al.*, 1987).

The crystal structure is rich in several types of supramolecular contacts, particularly strong charge-assisted hydrogen bonds (N^+^—H···O^-^) and weaker short distance contacts, namely C—H···F, F···F and C—H···π interactions (see Table 2 for a full listing and division into families). Hydrogen bonds appear between the two charged species comprising the asymmetric unit, establishing direct connections between the positively charged ammonium cation and the ketonic oxygen atoms (with partial negative charge): N1 donates H1*Z* in a bifurcated hydrogen bonding interaction simultaneously to O1 and O2; this leads to the formation of two spiral chains with graph set motif C_2_^1^(4) (Grell *et al.*, 1999), with the N1—H1*Z* bond being common to both. We note that the importance of these spirals in the crystal structure can be distinguished due to the asymmetric nature of the aforementioned bifurcated hydrogen bond: the main spiral chain has a considerably shorter N···O distance, being thus formed by the sequence H1X—N1—H1Z···O1 (dashed pink lines in Figs 2 and 3); the other supramolecular chain can be envisaged as formed by the sequence H1Y—N1—H1Z···O2 (dashed green lines in Figs 2 and 3). Noteworthy is that all these interactions are confined into a small hydrophilic space of the crystal structure which runs parallel to the *b* axis. The C—H···F contacts occur between H atoms from the aromatic rings and neighboring F atoms, being also close to the observed F···F contacts (dashed violet lines in Fig. 3). The C—H···π contacts arise from H atoms belonging to the terminal —CH_3_ moiety of the PrNH_3_^+^ cation, or from an aromatic ring, with the terminal ring of neighbouring naphthyl moiety. All the above mentioned supramolecular interactions contribute decisively for the crystal packing, in which the cations and anions are disposed into layers parallel to the (1 0 1) plane (Fig. 3).

### Experimental

All chemicals were purchased from commercial sources and used as received.

1-(2-Naphthoyl)-3,3,3-trifluoroacetone (1.06 g, 4.00 mmol) was dissolved in CHCl_3_ (20 ml) at ambient temperature. Propylamine (0.34 g, 4.00 mmol) was added and a yellow precipitate formed. The resultant mixture was further stirred for 16 h. After evaporation of the solvent to dryness, the resulting yellow solid was dissolved in CH_2_Cl_2_ at 40°C. Excess of dried MgSO_4_ was added to the solution, which was then filtered off and evaporated to dryness. Suitable crystals of the title compound were isolated by slow cooling to ambient temperature of a hot concentrated solution in CH_2_Cl_2_.

### Refinement

Hydrogen atoms bound to carbon were placed at their idealized positions and were included in the final structural model in riding-motion approximation with C—H = 0.95 Å (aromatic and methine C—H), 0.99 Å (—CH_2_—) and 0.98 Å (—CH_3_). Hydrogen atoms associated with the —NH_3_^+^ group were directly located from difference Fourier maps and were included in the final structural model with the N—H and H···H distances restrained to 0.95 (1) and 1.55 (1) Å, respectively, as to ensure a chemically reasonable geometry for this moiety. The isotropic thermal displacement parameters for hydrogen atoms were fixed at 1.2×*U*_eq_ (C—H and —CH_2_) or 1.5×*U*_eq_ (—CH_3_ and —NH_3_^+^) of the respective parent atoms.

The —CF_3_ group was found to be disordered and was modelled over two distinct positions with complementary rates of occupancy calculated from unrestrained refinement. The site occupancies ultimately converged to 0.830 (7) and 0.170 (7).

### Crystal data

**Table:**

C_3_H_10_N^+^·C_14_H_8_F_3_O_2_^−^	*F*(000) = 680
*M**_r_* = 325.32	*D*_x_ = 1.364 Mg m^−^^3^
Monoclinic, *P*2_1_/*n*	Mo *K*α radiation, λ = 0.71073 Å
Hall symbol: -P 2yn	Cell parameters from 4047 reflections
*a* = 16.1038 (11) Å	θ = 2.4–24.3°
*b* = 5.7008 (4) Å	µ = 0.11 mm^−^^1^
*c* = 17.2621 (11) Å	*T* = 150 K
β = 91.497 (4)°	Needle, colourless
*V* = 1584.20 (19) Å^3^	0.16 × 0.07 × 0.05 mm
*Z* = 4	

### Data collection

**Table:**

Bruker X8 KappaCCD APEXII diffractometer	2883 independent reflections
Radiation source: fine-focus sealed tube	1958 reflections with *I* > 2σ(*I*)
graphite	*R*_int_ = 0.068
ω and φ scans	θ_max_ = 25.4°, θ_min_ = 3.7°
Absorption correction: multi-scan (*SADABS*; Sheldrick, 1998)	*h* = −19→19
*T*_min_ = 0.982, *T*_max_ = 0.994	*k* = −6→6
21307 measured reflections	*l* = −20→20

### Refinement

**Table:**

Refinement on *F*^2^	Primary atom site location: structure-invariant direct methods
Least-squares matrix: full	Secondary atom site location: difference Fourier map
*R*[*F*^2^ > 2σ(*F*^2^)] = 0.050	Hydrogen site location: inferred from neighbouring sites
*wR*(*F*^2^) = 0.117	H atoms treated by a mixture of independent and constrained refinement
*S* = 1.06	*w* = 1/[σ^2^(*F*_o_^2^) + (0.0422*P*)^2^ + 1.0164*P*] where *P* = (*F*_o_^2^ + 2*F*_c_^2^)/3
2883 reflections	(Δ/σ)_max_ = 0.004
246 parameters	Δρ_max_ = 0.20 e Å^−^^3^
42 restraints	Δρ_min_ = −0.21 e Å^−^^3^

### Special details

**Table:**

Geometry. All e.s.d.'s (except the e.s.d. in the dihedral angle between two l.s. planes) are estimated using the full covariance matrix. The cell e.s.d.'s are taken into account individually in the estimation of e.s.d.'s in distances, angles and torsion angles; correlations between e.s.d.'s in cell parameters are only used when they are defined by crystal symmetry. An approximate (isotropic) treatment of cell e.s.d.'s is used for estimating e.s.d.'s involving l.s. planes.
Refinement. Refinement of *F*^2^ against ALL reflections. The weighted *R*-factor *wR* and goodness of fit *S* are based on *F*^2^, conventional *R*-factors *R* are based on *F*, with *F* set to zero for negative *F*^2^. The threshold expression of *F*^2^ > σ(*F*^2^) is used only for calculating *R*-factors(gt) *etc*. and is not relevant to the choice of reflections for refinement. *R*-factors based on *F*^2^ are statistically about twice as large as those based on *F*, and *R*- factors based on ALL data will be even larger.

### Fractional atomic coordinates and isotropic or equivalent isotropic displacement parameters (Å^2^)

**Table:**

	*x*	*y*	*z*	*U*_iso_*/*U*_eq_	Occ. (<1)
O1	−0.17996 (9)	−0.2046 (3)	0.67034 (9)	0.0292 (4)	
O2	−0.11872 (9)	0.1894 (3)	0.76308 (9)	0.0290 (4)	
C1	−0.08668 (15)	−0.3303 (5)	0.57660 (14)	0.0303 (6)	
C2	−0.10632 (14)	−0.1814 (4)	0.64723 (13)	0.0248 (6)	
C3	−0.04273 (14)	−0.0443 (5)	0.67711 (14)	0.0281 (6)	
H3	0.0101	−0.0584	0.6544	0.034*	
C4	−0.05040 (14)	0.1161 (4)	0.73921 (13)	0.0243 (6)	
C5	0.02702 (13)	0.2027 (4)	0.77986 (13)	0.0237 (5)	
C6	0.02518 (14)	0.4157 (4)	0.82202 (14)	0.0277 (6)	
H6	−0.0245	0.5056	0.8219	0.033*	
C7	0.09322 (14)	0.4941 (5)	0.86279 (14)	0.0286 (6)	
H7	0.0904	0.6381	0.8903	0.034*	
C8	0.16822 (14)	0.3635 (4)	0.86470 (13)	0.0233 (6)	
C9	0.17081 (13)	0.1478 (4)	0.82323 (13)	0.0209 (5)	
C10	0.09915 (13)	0.0741 (4)	0.78071 (13)	0.0231 (5)	
H10	0.1010	−0.0679	0.7520	0.028*	
C11	0.24530 (14)	0.0163 (4)	0.82469 (14)	0.0266 (6)	
H11	0.2476	−0.1278	0.7972	0.032*	
C12	0.31392 (15)	0.0937 (5)	0.86504 (14)	0.0305 (6)	
H12	0.3636	0.0039	0.8652	0.037*	
C13	0.31131 (15)	0.3064 (5)	0.90644 (14)	0.0312 (6)	
H13	0.3592	0.3585	0.9347	0.037*	
C14	0.24057 (14)	0.4381 (5)	0.90631 (14)	0.0277 (6)	
H14	0.2398	0.5813	0.9344	0.033*	
F1	−0.1330 (2)	−0.2618 (5)	0.51583 (13)	0.0712 (12)	0.830 (7)
F2	−0.1071 (3)	−0.5535 (4)	0.58622 (18)	0.0660 (12)	0.830 (7)
F3	−0.01008 (15)	−0.3235 (8)	0.5544 (2)	0.0829 (16)	0.830 (7)
F1'	−0.0653 (12)	−0.226 (2)	0.5185 (7)	0.059 (5)	0.170 (7)
F2'	−0.1435 (9)	−0.464 (4)	0.5531 (10)	0.069 (5)	0.170 (7)
F3'	−0.0244 (9)	−0.480 (2)	0.5937 (7)	0.052 (5)	0.170 (7)
N1	0.74144 (12)	0.3443 (4)	0.67812 (11)	0.0252 (5)	
H1X	0.7588 (12)	0.495 (2)	0.6613 (12)	0.038*	
H1Y	0.7898 (9)	0.262 (3)	0.6976 (12)	0.038*	
H1Z	0.7049 (10)	0.363 (4)	0.7203 (10)	0.038*	
C15	0.69841 (15)	0.2055 (5)	0.61567 (14)	0.0306 (6)	
H15A	0.6813	0.0525	0.6372	0.037*	
H15B	0.7380	0.1743	0.5740	0.037*	
C16	0.62297 (15)	0.3274 (5)	0.58129 (15)	0.0313 (6)	
H16A	0.5845	0.3637	0.6235	0.038*	
H16B	0.5940	0.2182	0.5451	0.038*	
C17	0.64185 (18)	0.5528 (5)	0.53828 (16)	0.0413 (7)	
H17A	0.6681	0.6653	0.5742	0.062*	
H17B	0.5901	0.6194	0.5168	0.062*	
H17C	0.6796	0.5190	0.4961	0.062*	

### Atomic displacement parameters (Å^2^)

**Table:**

	*U*^11^	*U*^22^	*U*^33^	*U*^12^	*U*^13^	*U*^23^
O1	0.0236 (9)	0.0286 (11)	0.0355 (10)	−0.0029 (8)	0.0063 (7)	−0.0031 (8)
O2	0.0247 (9)	0.0283 (11)	0.0341 (10)	0.0026 (8)	0.0030 (7)	−0.0044 (8)
C1	0.0254 (13)	0.0346 (18)	0.0307 (15)	−0.0062 (13)	0.0011 (11)	−0.0036 (13)
C2	0.0257 (13)	0.0239 (15)	0.0249 (12)	0.0036 (11)	0.0002 (10)	0.0027 (11)
C3	0.0218 (12)	0.0341 (16)	0.0285 (13)	−0.0013 (11)	0.0034 (10)	−0.0056 (12)
C4	0.0237 (12)	0.0230 (14)	0.0265 (13)	−0.0008 (11)	0.0022 (10)	0.0047 (11)
C5	0.0239 (12)	0.0223 (14)	0.0250 (12)	−0.0019 (11)	0.0028 (9)	−0.0010 (11)
C6	0.0252 (13)	0.0220 (15)	0.0359 (14)	0.0042 (11)	0.0024 (10)	−0.0025 (12)
C7	0.0334 (14)	0.0187 (14)	0.0336 (14)	0.0018 (11)	0.0005 (11)	−0.0059 (11)
C8	0.0274 (13)	0.0191 (14)	0.0234 (12)	−0.0018 (10)	0.0025 (10)	0.0001 (11)
C9	0.0246 (12)	0.0162 (14)	0.0222 (12)	−0.0022 (10)	0.0027 (9)	0.0024 (10)
C10	0.0282 (13)	0.0169 (14)	0.0243 (13)	−0.0018 (10)	0.0032 (10)	−0.0015 (11)
C11	0.0288 (13)	0.0216 (15)	0.0295 (13)	0.0030 (11)	0.0035 (10)	0.0004 (11)
C12	0.0243 (13)	0.0336 (17)	0.0337 (14)	0.0021 (11)	0.0013 (11)	0.0048 (13)
C13	0.0275 (13)	0.0354 (17)	0.0305 (14)	−0.0042 (12)	−0.0018 (10)	0.0014 (13)
C14	0.0319 (14)	0.0218 (15)	0.0295 (13)	−0.0039 (11)	0.0013 (10)	−0.0015 (12)
F1	0.105 (3)	0.072 (2)	0.0346 (13)	0.0386 (19)	−0.0269 (14)	−0.0150 (13)
F2	0.120 (3)	0.0212 (15)	0.0590 (18)	0.0016 (15)	0.0397 (18)	−0.0044 (12)
F3	0.0352 (13)	0.128 (4)	0.087 (2)	−0.0328 (17)	0.0303 (14)	−0.079 (3)
F1'	0.090 (10)	0.048 (7)	0.040 (6)	0.008 (7)	0.025 (6)	0.011 (5)
F2'	0.054 (7)	0.082 (10)	0.071 (8)	−0.035 (7)	0.018 (6)	−0.045 (7)
F3'	0.061 (8)	0.047 (7)	0.046 (6)	0.035 (6)	−0.010 (5)	−0.021 (5)
N1	0.0243 (10)	0.0228 (12)	0.0287 (11)	0.0008 (9)	0.0026 (9)	0.0017 (10)
C15	0.0380 (14)	0.0206 (15)	0.0331 (14)	−0.0017 (12)	−0.0006 (11)	−0.0032 (12)
C16	0.0358 (14)	0.0258 (16)	0.0320 (14)	−0.0041 (12)	−0.0034 (11)	−0.0015 (12)
C17	0.0560 (18)	0.0306 (17)	0.0368 (16)	0.0005 (14)	−0.0052 (13)	0.0049 (13)

### Geometric parameters (Å, °)

**Table:**

O1—C2	1.268 (3)	C9—C10	1.415 (3)
O2—C4	1.256 (3)	C10—H10	0.9500
C2—C3	1.377 (3)	C11—C12	1.364 (3)
C3—C4	1.417 (3)	C11—H11	0.9500
C1—F1'	1.223 (11)	C12—C13	1.408 (4)
C1—F2'	1.252 (12)	C12—H12	0.9500
C1—F3	1.302 (3)	C13—C14	1.364 (3)
C1—F2	1.325 (4)	C13—H13	0.9500
C1—F1	1.329 (3)	C14—H14	0.9500
C1—F3'	1.344 (10)	N1—C15	1.493 (3)
C1—C2	1.526 (3)	N1—H1X	0.952 (9)
C3—H3	0.9500	N1—H1Y	0.962 (9)
C4—C5	1.498 (3)	N1—H1Z	0.953 (9)
C5—C10	1.373 (3)	C15—C16	1.508 (3)
C5—C6	1.416 (3)	C15—H15A	0.9900
C6—C7	1.362 (3)	C15—H15B	0.9900
C6—H6	0.9500	C16—C17	1.518 (4)
C7—C8	1.418 (3)	C16—H16A	0.9900
C7—H7	0.9500	C16—H16B	0.9900
C8—C14	1.418 (3)	C17—H17A	0.9800
C8—C9	1.424 (3)	C17—H17B	0.9800
C9—C11	1.414 (3)	C17—H17C	0.9800
			
F1'—C1—F2'	104.5 (10)	C11—C9—C8	119.0 (2)
F1'—C1—F3	57.0 (8)	C10—C9—C8	118.9 (2)
F2'—C1—F3	127.9 (6)	C5—C10—C9	121.7 (2)
F1'—C1—F2	130.2 (6)	C5—C10—H10	119.2
F3—C1—F2	107.8 (3)	C9—C10—H10	119.2
F1'—C1—F1	51.3 (9)	C12—C11—C9	120.9 (2)
F2'—C1—F1	62.2 (11)	C12—C11—H11	119.6
F3—C1—F1	106.0 (3)	C9—C11—H11	119.6
F2—C1—F1	104.2 (3)	C11—C12—C13	120.2 (2)
F1'—C1—F3'	105.3 (9)	C11—C12—H12	119.9
F2'—C1—F3'	102.5 (11)	C13—C12—H12	119.9
F3—C1—F3'	51.4 (7)	C14—C13—C12	120.6 (2)
F2—C1—F3'	63.1 (7)	C14—C13—H13	119.7
F1—C1—F3'	139.4 (5)	C12—C13—H13	119.7
F1'—C1—C2	117.0 (6)	C13—C14—C8	120.7 (2)
F2'—C1—C2	115.7 (6)	C13—C14—H14	119.6
F3—C1—C2	115.8 (2)	C8—C14—H14	119.6
F2—C1—C2	112.1 (2)	C15—N1—H1X	113.1 (14)
F1—C1—C2	110.1 (2)	C15—N1—H1Y	110.5 (14)
F3'—C1—C2	110.3 (5)	H1X—N1—H1Y	107.8 (12)
O1—C2—C3	129.3 (2)	C15—N1—H1Z	109.1 (14)
O1—C2—C1	114.1 (2)	H1X—N1—H1Z	108.8 (13)
C3—C2—C1	116.6 (2)	H1Y—N1—H1Z	107.3 (12)
C2—C3—C4	124.8 (2)	N1—C15—C16	113.2 (2)
C2—C3—H3	117.6	N1—C15—H15A	108.9
C4—C3—H3	117.6	C16—C15—H15A	108.9
O2—C4—C3	123.9 (2)	N1—C15—H15B	108.9
O2—C4—C5	117.5 (2)	C16—C15—H15B	108.9
C3—C4—C5	118.6 (2)	H15A—C15—H15B	107.7
C10—C5—C6	118.8 (2)	C15—C16—C17	114.4 (2)
C10—C5—C4	121.5 (2)	C15—C16—H16A	108.7
C6—C5—C4	119.6 (2)	C17—C16—H16A	108.7
C7—C6—C5	121.3 (2)	C15—C16—H16B	108.7
C7—C6—H6	119.4	C17—C16—H16B	108.7
C5—C6—H6	119.4	H16A—C16—H16B	107.6
C6—C7—C8	120.8 (2)	C16—C17—H17A	109.5
C6—C7—H7	119.6	C16—C17—H17B	109.5
C8—C7—H7	119.6	H17A—C17—H17B	109.5
C14—C8—C7	122.8 (2)	C16—C17—H17C	109.5
C14—C8—C9	118.6 (2)	H17A—C17—H17C	109.5
C7—C8—C9	118.5 (2)	H17B—C17—H17C	109.5
C11—C9—C10	122.1 (2)		
			
F1'—C1—C2—O1	120.2 (11)	C4—C5—C6—C7	176.8 (2)
F2'—C1—C2—O1	−3.6 (13)	C5—C6—C7—C8	−0.4 (4)
F3—C1—C2—O1	−175.4 (3)	C6—C7—C8—C14	−180.0 (2)
F2—C1—C2—O1	−51.1 (4)	C6—C7—C8—C9	−0.2 (3)
F1—C1—C2—O1	64.4 (3)	C14—C8—C9—C11	−0.3 (3)
F3'—C1—C2—O1	−119.5 (9)	C7—C8—C9—C11	180.0 (2)
F1'—C1—C2—C3	−60.3 (11)	C14—C8—C9—C10	−179.1 (2)
F2'—C1—C2—C3	175.8 (13)	C7—C8—C9—C10	1.1 (3)
F3—C1—C2—C3	4.0 (4)	C6—C5—C10—C9	0.8 (3)
F2—C1—C2—C3	128.3 (3)	C4—C5—C10—C9	−175.8 (2)
F1—C1—C2—C3	−116.2 (3)	C11—C9—C10—C5	179.8 (2)
F3'—C1—C2—C3	60.0 (9)	C8—C9—C10—C5	−1.4 (3)
O1—C2—C3—C4	−4.8 (4)	C10—C9—C11—C12	178.8 (2)
C1—C2—C3—C4	175.9 (2)	C8—C9—C11—C12	0.0 (3)
C2—C3—C4—O2	−16.0 (4)	C9—C11—C12—C13	0.4 (4)
C2—C3—C4—C5	163.2 (2)	C11—C12—C13—C14	−0.4 (4)
O2—C4—C5—C10	154.1 (2)	C12—C13—C14—C8	0.1 (4)
C3—C4—C5—C10	−25.2 (3)	C7—C8—C14—C13	180.0 (2)
O2—C4—C5—C6	−22.4 (3)	C9—C8—C14—C13	0.2 (3)
C3—C4—C5—C6	158.2 (2)	N1—C15—C16—C17	65.1 (3)
C10—C5—C6—C7	0.1 (4)		

### Hydrogen-bond geometry (Å, °)

**Table:**

Cg is the centroid of the C8–C14 ring.

**Table:**

*D*—H···*A*	*D*—H	H···*A*	*D*···*A*	*D*—H···*A*
Strong hydrogen bonds—···..	.	.	.	.
N1—H1X···O1^i^	0.95 (1)	1.98 (1)	2.871 (3)	155 (2)
N1—H1Y···O2^ii^	0.96 (1)	1.88 (1)	2.798 (3)	159 (2)
N1—H1Z···O1^iii^	0.95 (1)	1.98 (1)	2.835 (2)	148.(2)
N1—H1Z···O2^iii^	0.95 (1)	2.34 (2)	2.985 (3)	124.(1)
C—H···F contacts—···..	.	.	.	.
C14—H14···F1^iv^	0.95	2.66	3.305 (4)	126
C13—H13···F1^iv^	0.95	2.69	3.320 (4)	124
C13—H13···F3^iii^	0.95	2.64	3.337 (4)	130
C—H···π contacts—···..	.	.	.	.
C11—H11···Cg^v^	0.95	2.93	3.677 (3)	136
C17—H17C···Cg^vi^	0.98	2.87	3.786 (3)	157
F···F contact—···..	.	.	.	.
C1—F3···F3^vii^	1.302 (3)	2.778 (6)	3.409 (4)	107.8 (2)

Symmetry codes: (i) *x*+1, *y*+1, *z*; (ii) *x*+1, *y*, *z*; (iii) −*x*+1/2, *y*+1/2, −*z*+3/2; (iv) *x*+1/2, −*y*+1/2, *z*+1/2; (v) *x*+1/2, −*y*+1/2, *z*−1/2; (vi) −*x*+1/2, *y*−1/2, −*z*+3/2; (vii) −*x*, −*y*−1, −*z*+1.
